# Predicting Visual Consciousness Electrophysiologically from Intermittent Binocular Rivalry

**DOI:** 10.1371/journal.pone.0076134

**Published:** 2013-10-04

**Authors:** Robert P. O’Shea, Jürgen Kornmeier, Urte Roeber

**Affiliations:** 1 Institute for Psychology, University of Leipzig, Leipzig, Germany; 2 Discipline of Psychology, School of Health and Human Sciences, Southern Cross University, Coffs Harbour, Australia; 3 Department of Psychology, University of Otago, Dunedin, New Zealand; 4 Cognitive Neuroscience Research Cluster, School of Health and Human Sciences, Southern Cross University, Coffs Harbour, Australia; 5 Institute for Frontier Areas of Psychology and Mental Health, Freiburg, Germany; 6 Department of Ophthalmology, University Eye Hospital, Freiburg, Germany; 7 Biomedical Sciences, School of Medical Sciences, The University of Sydney, Sydney, Australia; University of British Columbia, Canada

## Abstract

**Purpose:**

We sought brain activity that predicts visual consciousness.

**Methods:**

We used electroencephalography (EEG) to measure brain activity to a 1000-ms display of sine-wave gratings, oriented vertically in one eye and horizontally in the other. This display yields binocular rivalry: irregular alternations in visual consciousness between the images viewed by the eyes. We replaced both gratings with 200 ms of darkness, the *gap*, before showing a second display of the same rival gratings for another 1000 ms. We followed this by a 1000-ms mask then a 2000-ms inter-trial interval (ITI). Eleven participants pressed keys after the second display in numerous trials to say whether the orientation of the visible grating changed from before to after the gap or not. Each participant also responded to numerous non-rivalry trials in which the gratings had identical orientations for the two eyes and for which the orientation of both either changed physically after the gap or did not.

**Results:**

We found that greater activity from lateral occipital-parietal-temporal areas about 180 ms after initial onset of rival stimuli predicted a change in visual consciousness more than 1000 ms later, on re-presentation of the rival stimuli. We also found that less activity from parietal, central, and frontal electrodes about 400 ms after initial onset of rival stimuli predicted a change in visual consciousness about 800 ms later, on re-presentation of the rival stimuli. There was no such predictive activity when the change in visual consciousness occurred because the stimuli changed physically.

**Conclusion:**

We found early EEG activity that predicted later visual consciousness. Predictive activity 180 ms after onset of the first display may reflect adaption of the neurons mediating visual consciousness in our displays. Predictive activity 400 ms after onset of the first display may reflect a less-reliable brain state mediating visual consciousness.

## Introduction

A key quest for modern neuroscience is to determine whether patterns of activity in people’s brains can predict what they will see or do [1], [2], [3], [4], [5], [6]. We went on that quest, specifically to find early neural activity that predicts visual consciousness.

By visual consciousness we mean whether we see something, in which case we are conscious of it, or not, in which case we are not conscious of it, as happens in the phenomenon of binocular rivalry (see below).

By prediction, we mean whether specific patterns of brain activity, generalised over people, correlate with later instances or content of visual consciousness. (We are not attempting to predict from specific patterns of brain activity of one individual his or her later instances or content of visual consciousness.).

We searched for predictive patterns of brain activity by measuring the electrical activity of the brain non-invasively with scalp electrodes–electroencephalography (EEG). From the EEG data we calculated event-related potentials (ERPs) and their sources (using VARETA). ERPs are well-established and powerful techniques to study brain activity with high temporal resolution [7]. They are used in clinical diagnosis [8] and in basic research on phenomena such as visual search [9], attention [10], multistable perception [11], and consciousness [12]. VARETA is a powerful technique for locating sources of EEG activity that has some advantages over others [13].

We began our quest to find ERP/source activity that predicts visual consciousness on some paths made by others, which we review now.

### Intermittent Binocular Rivalry

**Figure 1:**
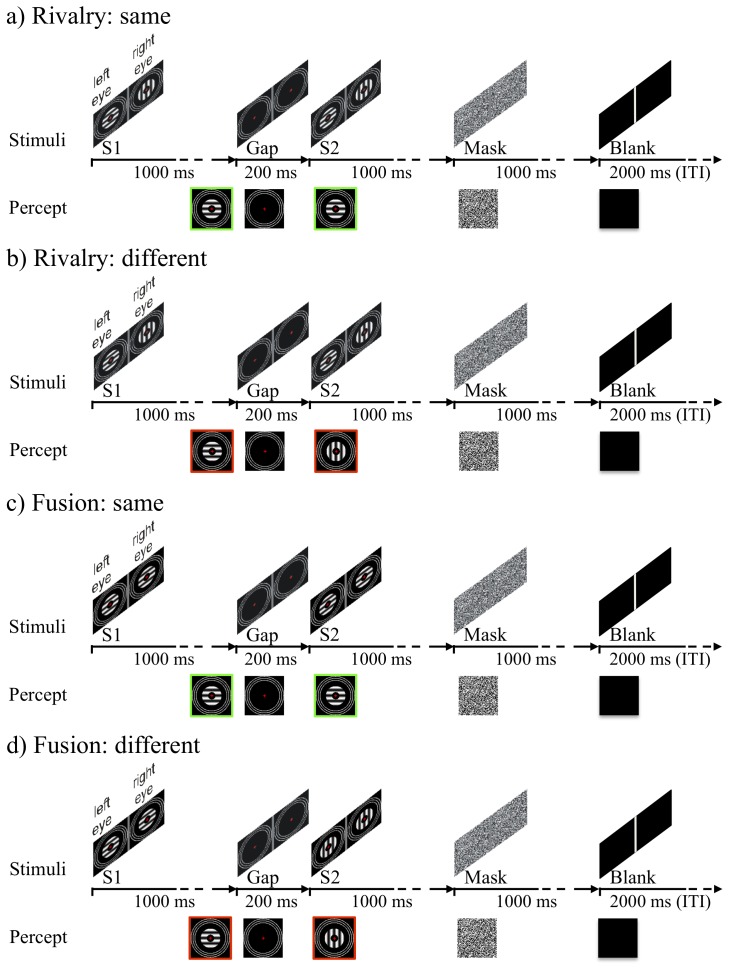
Illustrations of the sequence of events of some of the trials of the experiment along with the percepts, the visual consciousness, that might have accompanied those trials. Time runs from left to right in milliseconds. Note that this is not an exhaustive list of stimuli (whose orientations were counterbalanced over eyes for rivalry trials and displays for fusion trials) or of percepts. (a) A trial in which rival stimuli are presented and the percept is the same before the gap to after it. In this case, in the Stimuli panel, a horizontal grating is presented to the left eye, and a vertical grating is presented to the right eye for a first display of 1000 ms (S1), then there is a dark gap for 200 ms, then identical rival stimuli are presented for a second display of 1000 ms (S2), then there is a binocular, dotted mask for 1000 ms, then a dark, blank field for an inter-trial interval (ITI) for 2000 ms. Participants responded after the end of S2. In the Percept panel, in this case the participant sees the horizontal grating just before the gap, sees darkness (along with the vergence rings) during the gap, and sees the same horizontal grating after the gap (and then sees the mask and the blank field). (b) An identical rivalry trial to (a), except that the percept is different after the gap: from horizontal to vertical. (c) A trial in which fusion stimuli are presented and the percept is the same before the gap to after it. In this case, in the Stimuli panel, horizontal gratings are presented to both eyes, both in the first display before the gap and in the second display after the gap. The percept too is the same before and after the gap: in this case horizontal. (d) A fusion trial in which a change in the percept at the gap, from horizontal to vertical, occurs because the orientation of the fused gratings changes from horizontal before the gap to vertical after.

Those studying the neural correlates of visual consciousness [14], have typically simplified it using the fascinating bistable phenomenon of binocular rivalry [22], [23], [24]. This happens when one views different images continuously with each eye, such as the letter S to one eye and the letter A to the other [23], or a face to one eye and a house to the other [17], or a grating (e.g., vertical) to one eye and an orthogonal (i.e., horizontal) grating the other [25] (see Figure 1a). Immediately after onset of the images, one sees some combination of them for about 150 ms [26], [27], then one sees an S, or a house, or vertical, or whatever, with no trace of the other rival image for a second or so and then, sometimes after a brief period during which one does see some unstable composite of the two images, one sees the other image with no trace of first, again for a second or so, and then one sees the first image, and then the second image, and so on. That is, visual consciousness alternates randomly between two rival images, with no change in input to the two eyes, for as long as one cares to look. This property of rivalry makes it invaluable for investigating the neural correlates of consciousness [12], [28].

One problem for ERP research with using binocular rivalry to investigate the timing of neural processes predicting visual consciousness is the latency and variability of people’s reports of changes in visual consciousness. Reports lag by a mean of about 450 ms behind the change in visual consciousness [29] with a standard deviation of at least 10% [30], making reports unsuitable as the event for an ERP. Noest, van Ee, Nijs, and van Wezel (2007) [31], using a property of ambiguous figures discovered by Orbach, Ehrlich, and Heath (1963) [32], showed that if they interrupted a display of rival stimuli briefly with a dark field, this tended to prompt an alternation, thereby solving this problem. The interruption provides a clear event for the change in visual consciousness. This form of interrupted rivalry has become known as *intermittent binocular rivalry* [33].

Binocular rivalry is just one example of a range of perceptually bistable phenomena that can be destabilized (and stabilized) by interrupting displays for various times [32], [34]. Examples include the kinetic depth effect [35], quartet apparent motion [36], and the Necker cube [37]. It was with these latter two phenomena that the initial research was conducted into predicting, from brain activity, changes in visual perception with no change in the stimulus, reviewed next.

### Predicting Perceptual changes with Bistable Phenomena

As far as we are aware, research into predicting visual consciousness from brain activity began with quartet apparent motion. In this form of apparent motion, a “movie” repeatedly showing two frames is shown: one frame comprises two dark dots on the upper left and lower right corners of an imaginary rectangle and two light dots on the other corners; the other frame is the mirror image of the first. Apparent motion is bistable–sometimes the dots appear to move vertically and sometimes horizontally [36]. The event for EEG research is the onset of a frame. Müller, Federspiel, Fallgatter, and Strik (1999) repeatedly showed these two frames for one second each and asked participants to report the change in apparent motion [38]. They found an increase in EEG delta activity in the frame before that in which participants reported a perceptual alternation (i.e., about 550 ms before the alternation), interpreting this as an effect of decreased vigilance on visual consciousness–high vigilance, or sustained attention, tending to stabilise perception, presumably from top-down influences, low vigilance tending to allow perception to alternate.

The most popular phenomenon for researching EEG correlates of perceptual changes has been the Necker cube [39]. Kornmeier and Bach (2004, 2005, 2006) [40], [41], [42] measured EEG while repeatedly showing a lattice of ambiguous Necker cubes for 800 ms, followed by a dark field for 400 ms; participants reported when the 3D appearance of the cubes changed. They also had conditions in which the cubes were non-ambiguous by the addition of pictorial information, allowing them to compare the electrical activity of the brain after a change in an ambiguous display with that after a non-ambiguous display. They found ERPs that showed an occipital negativity about 250 ms after the onset of the cubes and a parietal positivity about 340 ms after onset that were larger when the 3D appearance changed than when it stayed the same; these were similar for ambiguous and non-ambiguous stimuli.

Britz, Landis, and Michel (2009) [43] looked before the onset of a Necker cube display for EEG activity that would predict changes in the 3D appearance. They conducted a complex topographical analysis showing greater activity in the right parietal region of the brain within 50 ms prior to the onset of the cube display that predicted a change in 3D orientation. They also compared ERPs for each electrode that discriminated between whether the 3D appearance of the cubes changed or stayed the same. Apart from finding similar patterns of differences after stimulus onset to those reported by Kornmeier and Bach, they showed two clusters of electrodes that predicted change, one about 75 ms before stimulus onset from left and frontal electrodes and the other about 25 ms before stimulus onset from left and parietal/occipital electrodes. There was no such predictive activity when the cubes were unambiguous.

Ehm, Bach, and Kornmeier (2011) [44] searched for EEG activity during the 200-ms gap prior to an intermittent display of Necker cubes. They found that modulation of gamma activity 200 ms before onset of the cubes predicted a change in the perceived 3D orientation. They explained this as reflecting a transient brain state of maximal instability preceding a perceptual reversal.

### Predicting Perceptual changes with Intermittent Binocular Rivalry

As far as we are aware, the first to predict a forthcoming binocular-rivalry alternation from brain activity were Britz, Pitts, and Michel (2011) [45]. They used similar methods and analyses to their research into the Necker cube [43], but with 600-ms displays of rival gratings differing between the eyes in orientation, colour, and spatial frequency, and separated by a dark gap of between 500 and 700 ms. During each dark gap, participants signalled with a key press the colour of the preceding percept. Britz et al. also included conditions in which a single stimulus was shown only to one eye for 600 ms, then changed after the gap to the other stimulus to the other eye. As in their study of the Necker cube [43] they found greater activity in the right parietal region of the brain within 50 ms prior to the onset of the rival stimuli that predicted a change in visual consciousness. In the same time range, they also found decreased activity in the occipital and temporal regions that predicted a change in visual consciousness. They found no predictive activity when visual consciousness changed because of a physical change in the stimuli. Britz et al. concluded that the right parietal region is causal in generating changes of visual consciousness, representing a top-down influence on sensory regions of the brain.

Hsieh, Colasb, and Kanwisher (2011) [46] looked for predictive activity with functional magnetic resonance imaging in a 2000-ms dark interval preceding discrete trials showing a 500-ms display of a face to one eye and a house to the other, a 1500-ms gap, and then a second display of the same rival stimuli. They asked participants to press keys during each presentation to report what they were seeing. Hsieh et al. also had otherwise identical trials in which the stimuli changed physically from the first to the second display (i.e., from both eyes’ viewing a face to both viewing a house or vice versa). Hsieh et al. defined regions of interest in the brain: the fusiform face area (FFA) and the parahippocampal place area. They found greater activity in the FFA that predicted a change in visual consciousness during rivalry by two seconds, concluding that this greater activity primed that area to win in a later rivalry competition.

We decided to use a similar procedure to Hsieh et al., of using discrete trials, rather than continually repeating displays of intermittent binocular rivalry as done by others. On each trial, we showed rival gratings for 1000 ms, then a dark gap for 200 ms, then a second display of identical rival gratings, then a dotted mask for 1000 ms, then an inter-trial interval of darkness for 2000 ms (Figure 1a,b). Discrete trials allowed us to collect participants’ reports on whether visual consciousness changed from the first to the second display after the second display had concluded. We included an equal number of trials in which the displays of the gratings were non-rival, fused (e.g., horizontal to both eyes) and either where the same in the second display (Figure 1c) or changed (i.e., vertical to both eyes; Figure 1d). We defined a region of interest, but also looked for marked activity outside that region of interest.

### Defining a Region of Interest (ROI)

In defining a region of interest (ROI), we were guided by two lines of research into binocular rivalry. One is a popular theory of binocular rivalry originated by McDougall in 1901 [47] involving reciprocal inhibition of neurons processing the rival images, adaptation of neurons processing the dominant image, and concurrent recovery of the previously adapted neurons of the suppressed image. The principles of this explanation exist in most models of rivalry [31], [48], [49], [50], [51], [52], [53], [54], [55], [56], [57], [58], [59], [60], [61].

There is now psychophysical evidence for the decline in the activity of the neurons processing the dominant rivalry stimulus after the onset of an episode of rivalry dominance and for an increase in the activity of neurons processing the suppressed stimulus. Alais, Cass, O’Shea, and Blake (2010) [29] found that sensitivity to contrast increments of the dominant rival stimulus is high immediately after onset of an episode of suppression and declines until the end of that episode of suppression whereas it is the opposite for sensitivity to contrast increments of the suppressed stimulus.

McDougall’s theory is that rivalry alternations are mediated by changes in activity accompanying adaptation of low-level neurons processing the basic visual features of the dominant, visible stimulus. Hence, we focused on early, adaptation-related modulations of ERPs to grating stimuli from occipito-parietal electrodes.

The other line of research that guided our search for a ROI was into ERPs during binocular rivalry when the event is a change in the orientation of one of the rival gratings to be the same as the other grating [20], [62], [63], [64], [65]. This approach yields physically identical stimuli (i.e., a change in the orientation of one grating) that differ in visual consciousness: when the change happens in the dominant eye, the change is visible; when the change happens in the suppressed eye, the change is harder to see, if not invisible. This research has shown that the earliest differences in the ERPs happen about 80–250 ms after the physical change, such that voltages are larger (either more positive or more negative depending on the time) when the change is seen than when not. The location of sources of this difference is in the parietal-temporal-occipital region [20]. This research, along with McDougall’s theory, allowed us to define a spatiotemporal ROI among parieto-occipital electrodes from 80–250 ms.

Murray, Brunet, and Michel (2008) [66] warned that defining a ROI can mean that researchers miss other, theoretically important activity. Hence, we also looked outside our ROI for marked predictive activity.

In our initial analyses, we opted to stay as close as possible to the raw EEG data by using simple treatment such as averaging and subtraction to produce ERPs [40], [41], [42], [44], [46], rather than complex treatment [43], [67]. (We are happy to share our data with any researchers who request them.) Apart from this, the main difference in our approach from most others is that we used long-duration, discrete trials that allowed us to look much earlier than others for predictive activity.

We found early predictive activity within our ROI about 180 ms after the onset of the first display of the rival stimuli. It was a bigger first negative deflection of the ERP when consciousness changed subsequently than when not. We also found unexpected, marked, predictive activity outside our ROI, about 400 ms after the onset of the same stimuli from parietal, central, and frontal electrodes. It was less positive voltage in the ERP when consciousness changed than when not. There was no such predictive activity from non-rival, fusion conditions. (We have previously reported this in published abstracts [68], [69].).

## Methods

### Ethics Statement

The study was performed in accordance with the ethical standards laid down in the Declaration of Helsinki [70] and with the ethics guidelines of the German Association of Psychology [71]. Ethical approval was granted by the German Research Foundation (DFG). We obtained written informed consent from each participant.

### Participants

Sixteen students from University of Leipzig volunteered for the experiment either for course credit or payment (€6 per hour). This reduced to 11 after we discarded two for not having normal or corrected-to-normal visual acuity in each eye as tested by the Freiburg Visual Acuity Test [72], one for whom we were unable to measure any usable EEG data, and two because there were too few trials (less than 1%) on which perception changed at the second display of rivalry stimuli. Mean age of the 11 participants (all right-handed; four male) was 27 years (range: 20 to 55 years).

The 11 included participants showed normal binocular rivalry during two 3-minute test sessions. We tested binocular rivalry with the same stimuli we used in the experiment, presented continuously. Participants pressed one key whenever and for as long as one grating was exclusively visible and another key whenever and for as long as the other grating was exclusively visible. We defined normal as the distributions of exclusive visibilities from the left and right eyes’ being monomodal, showing positive skew, and with similar modes and variabilities [73], [74], [75], [76], [77], [78], [79], [80].

The included participants also showed normal binocular and monocular visual evoked potentials. We tested visual-evoked potentials (VEPs) with black (0.8 Cd/sq m) and white (33.4 Cd/sq m) 5-deg checkerboards (0.5-deg checks) on a grey background (7.8 Cd/sq m). The checkerboards phase-reversed at 2 Hz. We tested 100 changes in the left eye (taking 50 s), 100 changes in the right eye, 100 changes in both eyes, then we repeated these blocks in the reverse order. We defined normal as the VEPs’ showing a N75 (C1), a P100 (P1), and a N175 (N2), that did not differ markedly between the eyes and that were larger for binocular stimulation than for monocular stimulation [7].

### Apparatus

Stimuli were presented on a ViewSonic Graphics Series G90fB, 19-inch, colour monitor showing 1024 pixels horizontally x 768 pixels vertically at 75 Hz. Participants viewed these stimuli through a Screenscope SA-200-Monitor-Type, four, front-surfaced mirror stereoscope attached to a chin rest. Viewing distance was 57 cm. They responded via a four-button keypad.

### Stimuli

Annulus-shaped patches of achromatic, vertical and horizontal, sine-wave gratings served as stimuli. The outer diameter of the annulus was 1.65 deg of visual angle; the inner diameter was 0.67 deg. Spatial frequency was 3.47 cycles/degree. The luminance of the gratings was 7.1 Cd/sq m with a Michelson contrast of 0.98.

The inner part of the stimuli contained a red, binocular fixation cross with vertical and horizontal arms 0.48 deg long and 0.06 deg thick. Outside the stimuli there were three concentric, equally spaced white rings of 0.03 deg thickness. The inner ring had a diameter of 2.50 deg; the outer ring had a diameter of 3.26 deg. These rings served to lock binocular vergence.

All stimuli were viewed on a dark background (0.4 Cd/sq m).

We used two types of stimuli;

1. Binocular *rivalry* stimuli: Each eye viewed either a vertical or a horizontal grating; the other eye viewed an identical grating of the orthogonal orientation.
2. Binocular *fusion* stimuli: Both eyes viewed identical gratings that were either vertical or horizontal.

### Procedure

We began by training each participant to use the keypad for the experiment proper. These training blocks consisted of 20 trials of fusion stimuli. Each trial consisted of:

1. a first display of the gratings, the rings, and the fusion crosses for 1000 ms,
2. followed by a gap during which only the fusion rings and the fixation crosses were displayed for 200 ms,
3. followed by a second display of everything for 1000 ms,
4. followed by a square 1.65 deg display of randomly black and white, one-pixel dots with the fixation cross for 1000 ms, and
5. finally a dark inter-trial-interval (ITI) with only the fixation crosses for 2000 ms.

Half the trials showed vertical gratings before the gap; the others showed horizontal gratings before the gap. On half of each set of trials, the orientation stayed the same after the gap; on the remaining trials, the orientation changed after the gap. Order of these practice trials was completely randomized afresh for each set of trials for each participant.

At the end of each trial participants pressed one of four keys, arranged in a two-by-two, square matrix, to record their visual consciousness before and after the gap. If a participant saw vertical after the gap, he or she chose between the leftmost two keys; if a participant saw horizontal after the gap, he or she chose between the rightmost two keys. If the perceived orientation had changed after the gap, the participant pressed the upper key; if the perceived orientation remained the same after the gap, the participant pressed the lower key. We explained this until the participant understood that he or she was to press a single key to communicate his or her perception of two properties: whether the gratings were the same or different orientation in the first and second display, and the orientation of the second display.

Usually participants achieved more than 90% correct on the first training block, but they could choose to have a second (or third, and so on) training block. Most participants trained for one block; no participants required more than three blocks. Participants pressed the response key during the display of dotted mask or during the ITI. If participants had not responded by the end of the ITI, then no response was recorded.

After completing the training participants went onto the experimental blocks, each comprising 40 trials. Half the trials were the same as the practice trials, involving fusion stimuli. The remaining trials were rivalry trials. In half of these, we presented vertical to the right eye and horizontal to the left eye; in the remainder it was the opposite. We alternated whether the first trial in each block was fusion or rivalry for each participant. We counterbalanced this over participants. Other than that, order of experimental trials was completely randomized afresh, without replacement, for each block for each participant.

One block of experimental trials took 3 minutes 30 s. The instructions were the same as for the practice trials except we warned participants that they might see some displays (i.e., the rivalry stimuli) that looked like a mixture of orientations. We asked them to pay attention to the predominant orientation before and after the gap in making their responses but not to press any key if both orientations were equally visible. We give schematic versions of rivalry and fusion trials in Figure 1.

Participants performed 16 blocks. We visited the participant after the first, fourth, and then every fourth block. No participant had any problems with the task, all getting more than 90% correct in the fusion trials of the first block.

### Electrophysiological Data

**Figure 2:**
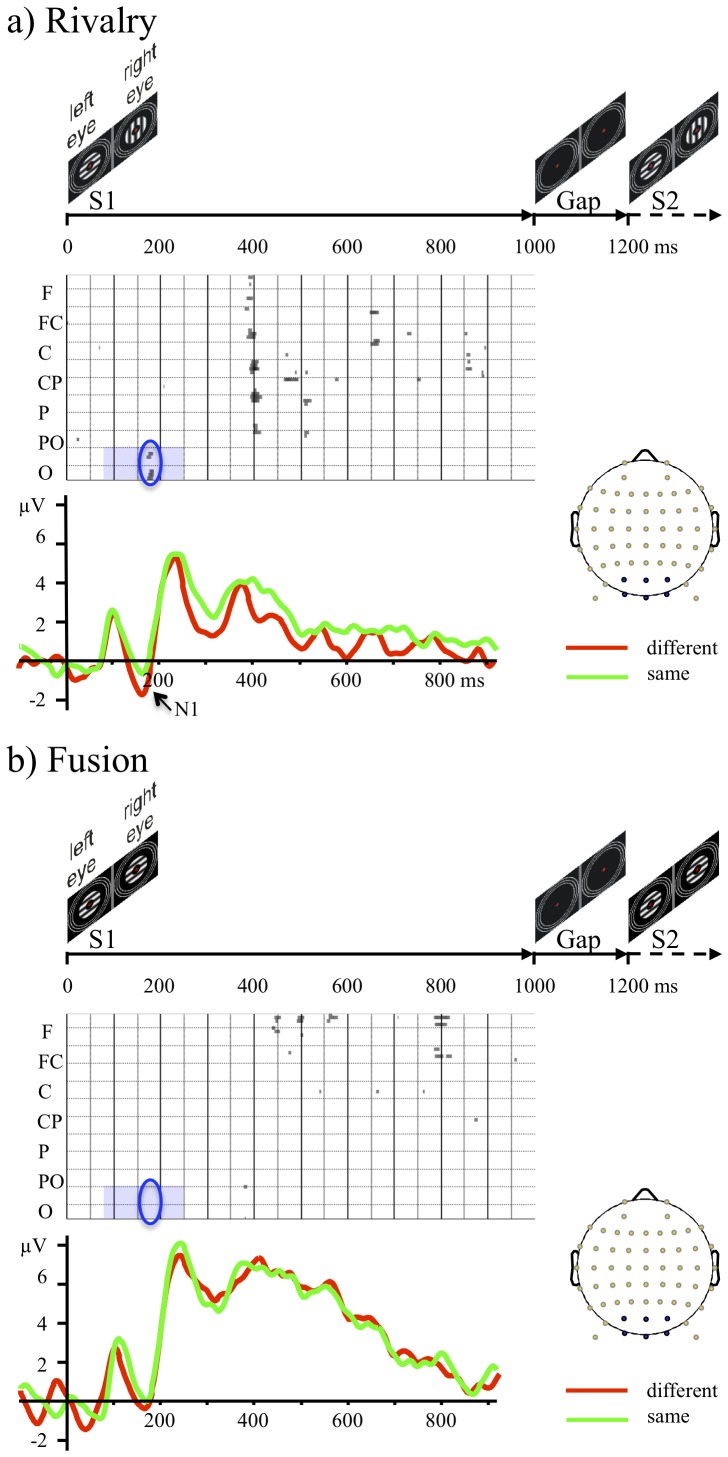
Illustration of events and event-related potentials (ERPs). (a) Top panel: schematic representation of the rivalry stimuli as a function of time for the first 1200 ms of a trial. Middle panel: *t* values for the difference in the voltage in the first display (S1) between trials when consciousness changed at the second display (S2) with those when it did not change, from 11 participants. *t* values, plotted as points whose density is proportional to *t* thresholded at *p* = .01, are from all electrodes (arrayed on the y axis from frontal, F, at the top to occipital, O, at the bottom) as a function of time (on the x axis). The region of interest (ROI) is the blue rectangle; the critical differences are enclosed by a blue oval. A cluster of electrodes shows predictive activity 180 ms after the onset of the stimuli. There is also noticeable predictive activity 400 ms after onset of the rival stimuli from clusters of parietal, central, and frontal electrodes. Lower panel: Event related potentials (ERPs), from a cluster of six parieto-occipital and occipital electrodes. ERPs are red for when consciousness changed and green for when it stayed the same. The ERPs show that the predictive activity within the ROI is in the first main, negative deflection, the N1 (arrowed). Its amplitude is greater when consciousness changed later, after the gap. There is also an enduring positivity from about 300 ms to 800 ms after onset that shows predictive activity. Its amplitude is less when consciousness changed later, after the gap. (b) Similar panels for fusion stimuli showing no predictive activity (middle panel) and essentially identical ERPs (lower panel).

We recorded EEG continuously with a BrainAmp system (Brain Products GmbH, Munich) using 66 active Ag/AgCl electrodes (actiCap). Four of them were for horizontal and vertical electrooculograms (EOGs), two were on the ear lobes. The remaining electrodes were mounted in an elastic cap in positions based on the modified 10–20 system [81] (Figure 2 depicts a schematic head with the electrode positions used). We used electrode FCz for a reference and electrode AFz for ground. The sample rate of EEG and EOGs was 500 Hz. We re-referenced the EEG data offline to the linked earlobes. We did this to allow comparison with earlier work on multistable visual phenomena that also used linked ears [40], [41], [42], [43], [64], [65], [82], [83]. We applied a 0.3–35 Hz bandpass filter (Kaiser windowed sinc FIR filter, 1857 points, Kaiser window beta 5.65326) to the data before analysis.

We calculated event-related potentials (ERPs) by averaging voltages separately for the four different events representing the factorial combination of stimuli (rivalry vs fusion) and whether visual consciousness changed after the gap, or stayed the same. We used a 2100-ms window, time-locked to the onset of the first display of gratings, including a baseline from minus 100 to 0 ms. For fusion trials, we included only trials with a correct response. Prior to averaging, we rejected any epoch containing a signal change of more than 60 µV at any EOG electrode and more than 150 µV at any EEG electrode by using an automatic peak-to-peak voltage artefact detection method.

To analyse neural generators, we used VARETA [20], [84], [85]. VARETA reconstructs sources by finding a discrete spline-interpolated solution to the EEG inverse problem: estimating the spatially smoothest intracranial primary current density (PCD) distribution compatible with the observed scalp voltages. This allows for point-to-point variation in the amount of spatial smoothness and restricts the allowable solutions to the grey matter (based on the probabilistic brain tissue maps available from the Montreal Neurological Institute [86]). VARETA constructs a 3D grid of 3,244 voxels, each of 7 mm per side, representing possible sources of the scalp potential, and registers the 59 informative scalp electrodes to the average probabilistic brain atlas. Then it constructs statistical parametric maps (SPMs) of the PCD estimates using a voxel-by-voxel Hotelling *T* ^2^ test against zero. We tested for predictive activity with a repeated-measures ANOVA comparing solutions for when visual consciousness changed with when it did not. We constructed corresponding SPMs based on the ANOVA’s output. For all SPMs, we used random field theory [87] to correct activation threshold for spatial dependencies between voxels. We show results in average, glass brains.

## Results and Discussion

### Behavioural Data

In the fusion trials, we had real, physical changes in the orientation of the stimuli from the first to the second interval. These allow us to assess response accuracy. Mean accuracy in the fusion trials was 94% with a standard deviation of 7%–high accuracy.

In the rivalry trials, the mean percentage of trials (standard deviation) in which perception changed was 37% (14%). Perception stayed the same in 57% (19%) of trials; there was no response in 6 (10%) of the rivalry trials.

### Electrophysiological Data within the ROI

After artifact rejection, the behavioural responses yielded a mean of 107 trials per participant for the rivalry/changed-consciousness events (standard deviation of 47), 148 (47) trials for the rivalry/same-consciousness events, 127 (29) trials for the fusion/changed-consciousness events, and 125 (28) trials for the fusion/same-consciousness events.

We show ERPs in two ways. One is by calculating, for each electrode at the sampling rate of 500 Hz, uncorrected *t*-tests on the difference in average voltage between trials in which visual consciousness changed and those when it did not change, as done by Britz et al. (2009, 2011) [43], [67]. The other is to average voltages over clusters of electrodes.

We show the results of the *t*-tests for rivalry stimuli in the middle panels of Figure 2a and 1b, where the electrodes are arranged from frontal (F; at the top) through fronto-central (FC), central (C), centro-parietal (CP), parietal (P), parieto-occipital (PO), to occipital (O; at the bottom). The density of the dots is determined by the value of *t*, thresholded at *p* = .01. We give a longer time sample, to 2000 ms after onset of the first display, in Figure S1 for rival stimuli and in Figure S2 for fusion stimuli (we discuss these in Item S1). The earliest neural correlates occurred within our ROI–the parieto-occipital and occipital electrodes about 180 ms after the onset of the first gratings. These represent 34 *t*-tests at *p*<.01. Because activity on one electrode at one time is neither independent of that on neighbouring electrodes or of earlier activity on the same electrode, we took four steps to assure ourselves that this activity is more than Type-I error:

1. We compared the number of significant *t*-tests, 34, in the ROI with the theoretical Type-I error rate assuming independence. With 10 electrodes and 85 samples between 80 and 250 ms, the expected number under the null hypothesis is 8.5. The obtained number is significantly greater than expected, χ^2^(1) = 77.27, *p*<.0001.
2. We looked at the number of significant *t*-tests from the fusion condition in the ROI. Any such significant tests *must* represent Type-1 error, otherwise it would mean we can predict from participants’ brain activity what the computer later chose to display, which is impossible! There were zero such significant tests within the ROI.
3. We performed running permutation tests. These yielded 46 significant tests (at *p*<.01) with the ROI in the rivalry conditions and none in the fusion conditions.
4. We took the advice of Lieberman and Cunningham (2009) [88] and replicated our finding in another project in which we tested McDougall’s theory by manipulating adaptation with flash suppression. We found the same difference in voltages after onset of a monocular, flash-suppression stimulus that predicted a rivalry alternation about one second later in a gap between two short displays of identical rivalry stimuli [89], [90].

In the bottom panel of Figure 2, we show mean ERP traces when visual consciousness changed after the gap (red line) and for when visual consciousness stayed the same after the gap (green line) (averaged across six parieto-occipital electrodes as shown on the schematic head). We see a trough of activity at 180 ms in the rivalry condition but not in the fusion condition: the well-known N1 component. We analysed the ERP data with an analysis of variance on average voltage for each participant between 170 ms and 190 ms; we give the ANOVA summary in Table S1. Critically the average deflection from baseline in voltage at 180 ms during rivalry is bigger–more negative–when there was a change in visual consciousness from the first to the second display than when there was no change, *F*(1, 10) = 7.38, *p* = .022, partial η^2^ = .42. There is no such predictive activity from fusion conditions, *F*(1, 10) = 0.42, *p* = .53, partial η^2^ = .04 (Figure 2b, Figure S2, Table S1). The absence of predictive activity from fusion conditions suggests that the predictive activity during rivalry arises from processing of the rival stimuli and not from the changes in perception. As far as we are aware, the enhanced negativity 180 ms after the onset of rivalry stimuli is the earliest ERP activity that predicts visual consciousness.

**Figure 3:**
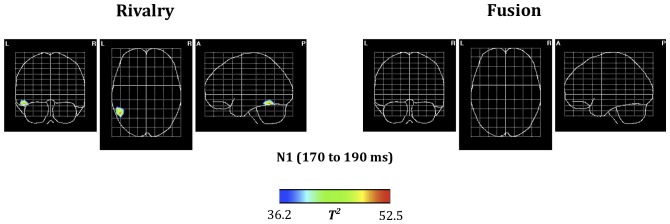
Source localisation by VARETA. Significant voxels (thresholded to *p*<.01, Bonferroni corrected) are shown in glass brains for the difference in sources between when visual consciousness changed from the first to the second displays and when visual consciousness stayed the same from the first to the second displays. The left panel shows rivalry conditions; the right panel shows fusion conditions. There is a significant difference only in the left lateral occipito-temporal/inferior temporal gyrus region for rivalry conditions.

We used VARETA to search for neural generators of activity from 170 to 190 ms. In Figure 3 we show the voxels whose activity differs significantly (thresholded to *p*<.01, Bonferroni corrected) between trials in which visual consciousness stayed the same from the first to the second displays and trials in which visual consciousness changed from the first to the second displays. Figure 3 shows a significant source only in the left lateral occipio-temporal gyrus and the inferior temporal gyrus for rivalry conditions. This agrees with other resesarch into the source of the visual N1 [91] and with other research into the sources of early EEG activity in binocular rivalry [20], [64].

### Electrophysiological Data Outside the ROI

Looking outside the ROI, we could not help but see a major difference in clusters of parietal, central, and frontal electrodes about 400 ms after the onset of the first rivalry display (middle panel of Figure 2a). The ERP traces (bottom panel of Figure 2a) show a prolonged positivity from about 300 ms to about 800 ms that is smaller when consciousness subsequently changed than when not. Although this difference is not significant in the parieto-occipital electrodes from which the traces in Figure 2 are averaged, it is significant in a cluster of central electrodes (Figure S1, Table S2), *F*(1, 10) = 11.02, *p* = .008, partial η^2^ = .52 (for data from 380 ms to 420 ms). There is no such activity in fusion conditions, *F*(1, 10) = 0.47 *p* = .508, partial η^2^ = .05. As far as we are aware no one else has reported this sort of predictive activity either.

## General Discussion

We found two main clusters of activity predicting an upcoming change in conscious perception of an unchanged binocular rivalry stimulus. The earlier one occurred in our ROI–occipital and parieto-occipital electrodes about 180 ms after the onset of the first display. Greater activity in the brain recorded from those electrodes was more likely to result in a change in visual consciousness 1020 ms later, at onset of the second display of identical rival stimuli. The source of this activity is the left, parietal-occipital-temporal region of the brain.

The later cluster of predictive activity occurred in parietal, central, and frontal electrodes about 400 ms after onset of the first display in which less activity predicted the later change in consciousness. We concede that, because this predictive activity is outside our ROI, we need to be cautious about its reliability.

We are not sure why the source we found for the early activity was confined to the left hemisphere; this is rather different from other studies finding consciousness-related activity predominantly in the right hemisphere [14], [20], [21], [45]. It is possible our source was significant only in the left hemisphere because we asked our participants to remember the visible orientation in both displays for more than two seconds; this may have set up some form of recurrent processing with the language areas of the brain in the left hemisphere to enable verbal encoding of the orientations. On the other hand, the absence of activity in the right hemisphere might represent a Type-II error from the heavy discounting by Bonferroni correction [92]. We note that the isolated left and right hemispheres can yield the experience of binocular rivalry [93], [94], [95] and that others have found bilateral occipital activity that predicts rivalry content [45].

### Proposed Explanation of the Predictive Activity

Greater initial activity might seem at first to be similar to greater fMRI activity found by Hsieh, Colasb, and Kanwisher (2011) [46] in the FFA that predicts by two seconds visual consciousness during binocular rivalry between an image of a face and an image of a house. However the activity found by Hsieh et al. was from non-rival conditions, while participants were looking at a dark field. Hsieh suggested that random increases in existing activity in the FFA predispose it to win the competition between later rivalry stimuli. Our activity, on the other hand, is from a first display of rivalry stimuli, during which the neurons are processing the same stimuli as in the second, to-be-predicted display of rival stimuli. So greater activity in our ROI predispose the neurons producing that activity to *lose* the rivalry competition in the second display.

Alais et al. (2010) [29] found psychophysical evidence they argued shows that low activity in the neurons processing the dominant stimulus presages a reversal of activity. At first blush, this might imply that any early predictive activity we found would be lower voltages just before the gap, but our predictive activity is higher voltages that are much, much earlier, just after the onset of the first display of rival stimuli at a time at which rivalry would just have been resolved [26], [27].

Critically, one property of neural adaptation is that when activity is high, decline of neural responsiveness is rather rapid, whereas when activity is low, decline of neural responsiveness is rather slow, if it is not even an increase [96], [97]. This explains why initial high activity predicts later visual consciousness: vigorous activity leads to substantial adaptation that would prompt an alternation at the gap. It is also consistent with long-term suppressive activity from similar orientations found psychophysically from other techniques [98].

It is possible that the predictive activity we found 400 ms after onset of the rival stimuli in other electrodes reveals the decline of activity from adaptation that would prompt a later change in visual consciousness at the gap. But we concede that this is rather speculative for two reasons.

1. That predictive activity is not from the parieto-occipital electrodes. It is these that show the earlier predictive activity and these that the theory would hold responsible for initial processing the stimuli at onset of the second display.
2. The activity recorded from parietal, central, and frontal electrodes is likely to be very complicated to explain, being contributed to by feed-forward and feedback connections from many other neurons [99].

Nevertheless, there is brain-imaging evidence of the involvement of parietal, central, and frontal brain areas in binocular rivalry [14], [21] and in alternations of the Necker cube [11]. Moreover the predictive ERP activity around 400 ms could be the peak of a long positivity found by Niedeggen, Wichmann, and Stoerig (2001) [100] from an ERP study of change blindness. They found this component when participants noticed a change in one of two complicated pictures–there was a greater positive deflection of the ERP between 300 and 700 ms after stimulus onset than when participants did not notice the change. This has become known as the “late positivity” [101], and is most pronounced from parietal and temporal electrodes.

Railo et al. [101] have attributed the late positivity to the application of attention and working memory to the stimulus. This is certainly consistent with our task that required participants to remember the orientation of the first display. It is important to note that our activity is *predictive* activity and not the classic late positivity. But if it is a similar component, then it is tempting to conclude that lower levels of such activity mean that the percept was not as well-established as when there are higher levels of such activity, as proposed by Kornmeier and Bach (2009) [83] from an EEG study of perceptual alternations between two-dimensional ambiguous figures. Perhaps a poorly established percept is more likely to change at the gap, whereas a well-established percept is more likely to endure at the gap to be seen in the second display.

### Relation to other Work Finding Predictive Activity with Multistable Displays

If predictive delta activity for quartet apparent motion is because decreased attention allows an alternation [38] then this seems different from binocular rivalry because decreased attention tends to decrease the rate of alternations [102], [103], [104], [105]. It is also different from the Necker cube because withdrawing attention decreases reversals in it [106]. Predictive gamma activity for alternations of the 3D orientation of the Necker cube [44] came from a 200-ms gap in a display. This means that, unlike in our study, the neurons were not processing the multistable stimulus when they yielded the predictive activity.

Predictive ERP activity 50 ms prior to a binocular rivalry display that yielded different visual consciousness from earlier displays [45] also must have arisen when the neurons responsible were processing the darkness of the gap between rival displays. Predictive greater fMRI activity in FFA that precedes visual consciousness of a face during rivalry [46] is the opposite of adaptation. There is no conflict with our results because this predictive activity came from the neurons when they were not currently processing rival stimuli, whereas ours came from neurons when they were processing rival stimuli.

### Relation to Theories of Visual Consciousness

Zeki and Bartels (1999) [107] proposed that visual consciousness is ubiquitous throughout the visual system, with each visual module, such as V4 processing colour, generating its own microconsciousness. In this theory, any binding that occurs between microconsciousness is simply fortuitous. Because our stimuli were achromatic, stationary gratings it is likely, according to Zeki and Bartels’s theory, that they were all processed within the same module: V1. It is not surprising therefore that early activity from parietal-occipital-temporal areas, nearby V1, might predict later consciousness for the same stimuli.

O’Regan and Noë (2001) [108] proposed that visual consciousness exists to allow one to explore the external environment by thinking about it, by preparing to move in it, or by acting on it. Their critical neural property, then must be involvement of motor areas. Our procedure is consistent with this theory, in that we asked our participants to assess the phenomenal contents of the first display of rivalry stimuli and to respond in different ways if the second display were the same or different from the first. Our results suggest that this assessment happens very fast, just after one has registered that there are two different stimuli, and not a grid. Of course, the predictive activity 400 ms after onset of the stimuli is also consistent with O’Regan and Noë’s theory in that it came from electrodes presumably recording from the motor cortex, among other areas. If so, this activity must be long-term preparation for a motor response, because none was made in our procedure until more than 1800 ms later.

Lamme (2010) [109] proposed that the critical neural operation for visual consciousness is recurrent processing. That is, he distinguished between:

- A feedforward sweep of activity generated by images falling on the retinas through V1 to V4 and into the dorsal pathway and ventral pathways to motor and frontal areas. It does not lead to visual consciousness.
- Recurrent processing in which activity from higher areas feeds back to maintain activity in lower areas. At the latest stages, it does lead to visual consciousness that is available for attentional scrutiny and verbal report.

Our procedure, requiring participants to attend to the rival stimuli and to report them, clearly requires processing within these latter stages. According to Lamme, our findings mean that processing within the feedforward sweep can predict visual consciousness mediated by recurrent processing. What is surprising about our finding is the timing.

The predictive activity we found occurs about 180 ms after the onset of the first display–inside the late parts of the feedforward sweep. However there is evidence that 180 ms is more than enough time for recurrent processing to be initiated. Foxe and Simpson (2002) [110] have estimated that the neural impulses flow from the eyes to prefrontal areas in about 80 ms. Moreover, they have adduced evidence that the early activity in ERPs, even within 50 ms after stimulus onset, involves recurrent processing. There is no necessary contradiction here if it is assumed that Lamme’s two stages operate in parallel. Perhaps what is surprising in the light of our results is just how late the predictive activity is, at the time of the second, and not the first (100 ms after an event) major ERP component to have been shown to be a neural correlate of consciousness in binocular rivalry [20], [64], [65] and in masking [111].

Dehaene and colleagues [112], [113], [114] proposed what has come to be known as neural global workspace theory [115]. Although Dehaene et al. agreed in general with Lamme’s notions of recurrent processing, they have emphasized the importance of recurrent processing’s creating a brain state involving sustained activation of thalamic, striate, extrastriate, cingulate, parietal, and frontal brain areas by synchronous oscillations in the gamma band [116]. Dehaene et al. have also emphasized the role of the fronto-parietal network for yielding access consciousness. One of their key ERP markers of conscious access of sensory information is the P300, which is supposed to occur during or after the creation of the global neural workspace by the synchronous oscillations [113]. Although we do not deny that the P300 is a reliable marker of consciousness, we do not see any predictive activity there in our recordings.

Based on the above discussion, one could speculate as follows: Effects of grating adaptation at the earliest levels of visual processing that could have been predictive, at the P1, 100 ms after onset of rival stimuli [20], [64], [65] either may not have been visible in our data because the spatial resolution of EEG is insufficient to distinguish activity of neurons processing the rival stimuli from neighbouring ocular dominance columns [117] or because rivalry was not completely resolved by then [26], [27].

The earliest predictive effects we find, the N1, 180 ms after onset of rival stimuli may reflect consequences of adaptation at an intermediate level of processing, once rivalry is established. Whether this arises from feedforward or recurrent processing cannot be decided from our data. In any case, according to Lamme (2010) [109] and Dehaene and colleagues [112], [113], [114] this predictive activity is likely pre-conscious.

The predictive effects in the late positivity [101], 400 ms after onset of the rival stimuli, may reflect processes mediating visual consciousness as defined by Lamme and Dehaene and colleagues. Its decreased amplitude before a change in visual consciousness compared to no change, may reflect reduced reliability of the processes mediating that consciousness [83].

## Conclusion

We set out to predict visual consciousness electrophysiologically using EEG. We found that greater activity in parietal-occipital-temporal areas about 180 ms after the onset of a first display of rivalry stimuli predicted a change in visual consciousness in a second display of the same stimuli by about one second. We also found that less activity in the neurons recorded from parietal, central, and frontal electrodes predicted a change in visual consciousness by about 800 ms. We propose that the predictive activity 180 ms after onset of the first display arises from high activity, leading to high adaptation, of the low-level neurons processing the initially visible rival stimulus. We propose that the predictive activity 400 ms after onset of the first display arises from low activity of a network of higher-level neurons mediating consciousness of the initially visible rival stimulus.

## Supporting Information

Figure S1 **Version of** **Figure 1a** **showing an additional 800 ms of the second display of rival stimuli and a second set of ERPs from a cluster of six central electrodes.** Top panel: schematic representation of the stimuli as a function of time. Middle panel: *t* values for the difference in the voltage between trials when consciousness changed at the second display with those when it did not change. These are from all electrodes (arrayed on the y axis from frontal, F, at the top to occipital, O, at the bottom) as a function of time (on the x axis). When the change in consciousness was from rivalry (a), there was a cluster of electrodes showing predictive activity 180 ms after the onset of the stimuli. About 400 ms after the onset of the first display of stimuli, there is other, widespread predictive activity. There is also other activity about 450 ms after the onset of the second display. It is more negative when consciousness changed after the gap. Lower panels: Average voltages, ERPs, from clusters of six parieto-occipital and occipital (OP) electrodes (see upper schematic head), and from six central electrodes (see lower schematic head), red for when consciousness changed and green for when it stayed the same. The upper ERPs, for parieto-occipital and occipital electrodes, show that the first predictive activity, in the first display of rival stimuli, was in the first main, negative deflection (the N1). They also show a prolonged positivity from 300 ms to 800 ms that is less when consciousness changed after the gap than when not. There is also a large difference between the two traces between about 300 ms and 800 ms after onset of the second display, with greater negativity when consciousness had changed after the gap. We discuss this in Item S1. The lower ERPs, for central electrodes, show that the predictive activity is only in the positivity in the first display, between 300 ms and 800 ms, maximal at 400 ms. There are also differences in the second display presumably arising from response preparation.
(PDF)

Figure S2 **Version of** **Figure 1b** **showing an additional 800 ms of the second display of fused stimuli.** Top panel: schematic representation of the stimuli as a function of time. Middle panel: *t* values for the difference in the voltage between trials when consciousness changed at the second display with those when it did not change. These are from all electrodes (arrayed on the y axis from frontal, F, at the top to occipital, O, at the bottom) as a function of time (on the x axis). There is no predictive activity in the first display. There are differences in the second display: higher voltages 100 ms and 450 ms after onset of different stimuli. Lower panel: Average voltages, ERPs, from clusters of six parieto-occipital and occipital (OP) electrodes, red for when consciousness changed and green for when it stayed the same. ERPs are essentially the same in the first display. In the second display, there are a bigger P1 and a bigger N1 when stimuli differed from the first display. There is also a late positivity from 300 ms to about 800 ms that is much bigger for different stimuli about 450 ms. We discuss these differences in Item S1.
(PDF)

Table S1 **Two-factor ANOVAs of average voltage from parieo-occipital (PO, O) electrodes for Rivalry and Fusion conditions from 170 to 190 ms after onset of the rival stimuli.**
(DOCX)

Table S2 **Two-factor ANOVAs of average voltage from central electrodes (fronto-central, FC, central, C, and centro-parietal, CP) for Rivalry and Fusion conditions from 380 to 420 ms after onset of the rival stimuli.**
(DOCX)

Item S1 **Discussion of activity in the second display.**
(DOCX)
