# Supplementary figures and images for: Predicting Visual Consciousness Electrophysiologically from Intermittent Binocular Rivalry
Source: PLoS One. 2013 Oct 4;8(10):e76134. doi: 10.1371/journal.pone.0076134 (PMC3790688; doi:10.1371/journal.pone.0076134)

# Rivalry

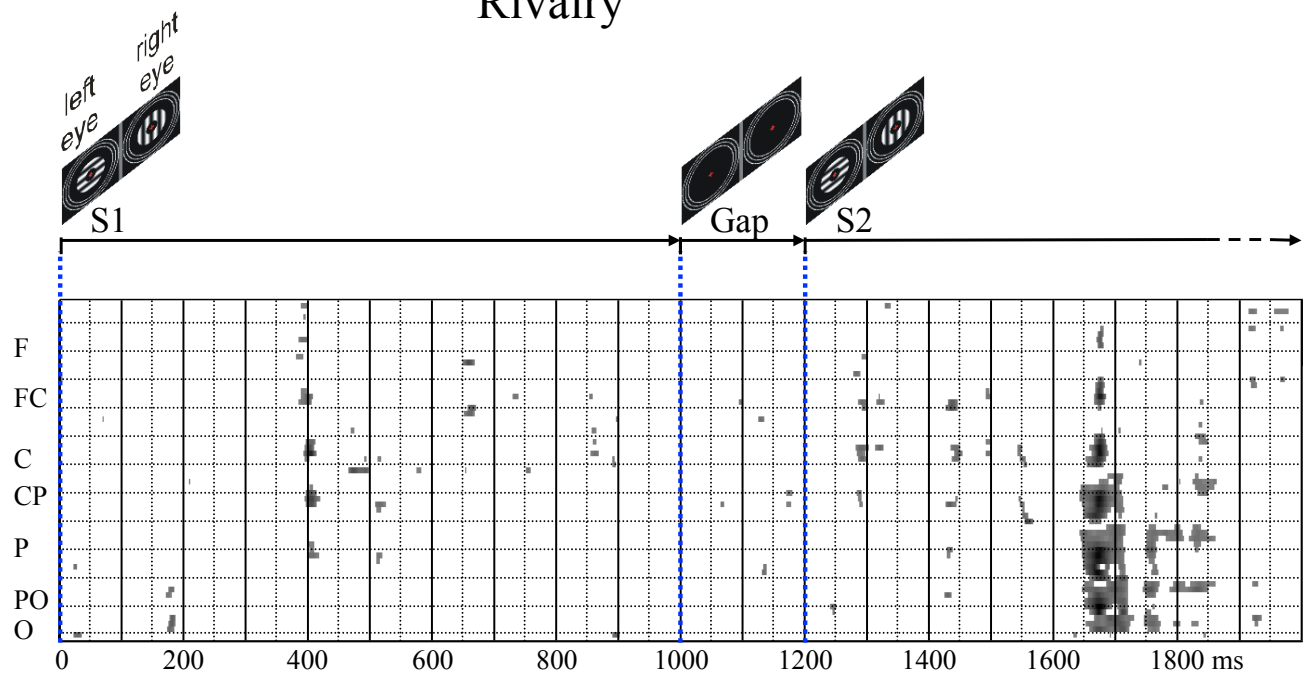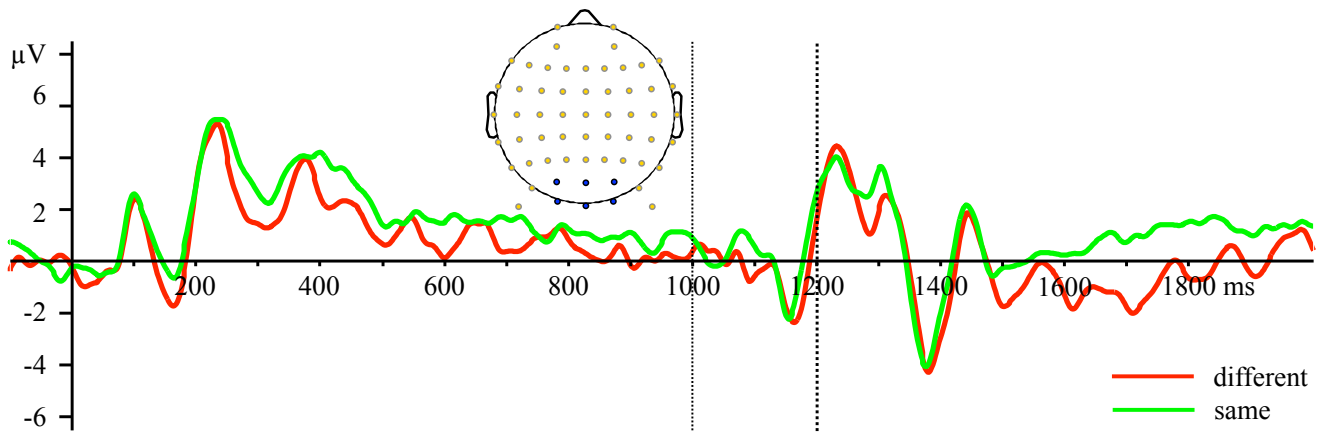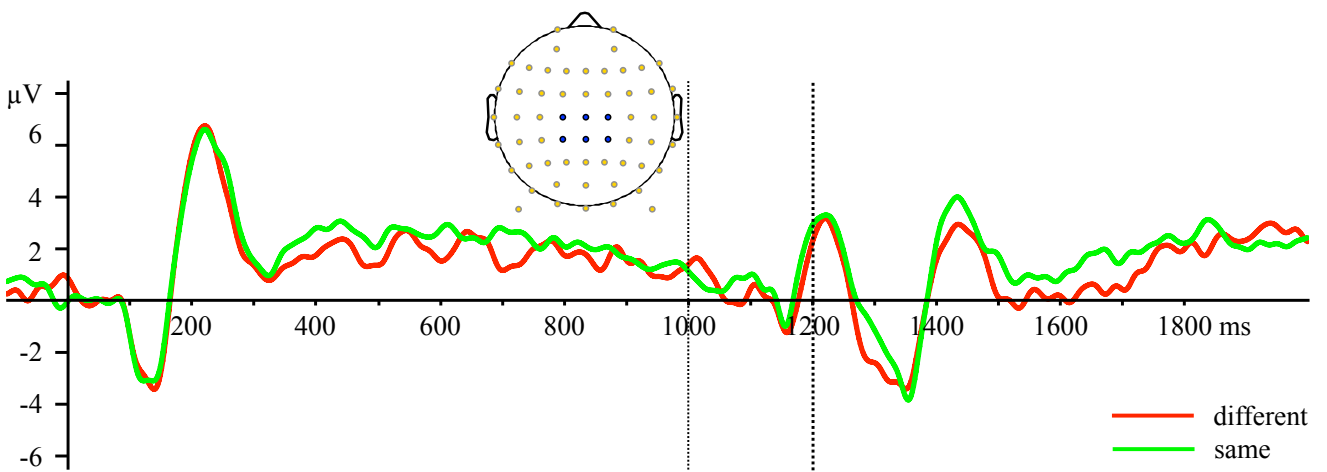

Supplement: Figure S1 — Version of Figure 1a showing an additional 800 ms of the second display of rival stimuli and a second set of ERPs from a cluster of six central electrodes. Top panel: schematic representation of the stimuli as a function of time. Middle panel: t values for the difference in the voltage between trials when consciousness changed at the second display with those when it did not change. These are from all electrodes (arrayed on the y axis from frontal, F, at the top to occipital, O, at the bottom) as a function of time (on the x axis). When the change in consciousness was from rivalry (a), there was a cluster of electrodes showing predictive activity 180 ms after the onset of the stimuli. About 400 ms after the onset of the first display of stimuli, there is other, widespread predictive activity. There is also other activity about 450 ms after the onset of the second display. It is more negative when consciousness changed after the gap. Lower panels: Average voltages, ERPs, from clusters of six parieto-occipital and occipital (OP) electrodes (see upper schematic head), and from six central electrodes (see lower schematic head), red for when consciousness changed and green for when it stayed the same. The upper ERPs, for parieto-occipital and occipital electrodes, show that the first predictive activity, in the first display of rival stimuli, was in the first main, negative deflection (the N1). They also show a prolonged positivity from 300 ms to 800 ms that is less when consciousness changed after the gap than when not. There is also a large difference between the two traces between about 300 ms and 800 ms after onset of the second display, with greater negativity when consciousness had changed after the gap. We discuss this in Item S1. The lower ERPs, for central electrodes, show that the predictive activity is only in the positivity in the first display, between 300 ms and 800 ms, maximal at 400 ms. There are also differences in the second display presumably arising f [file pone.0076134.s001.pdf]

# Fusion

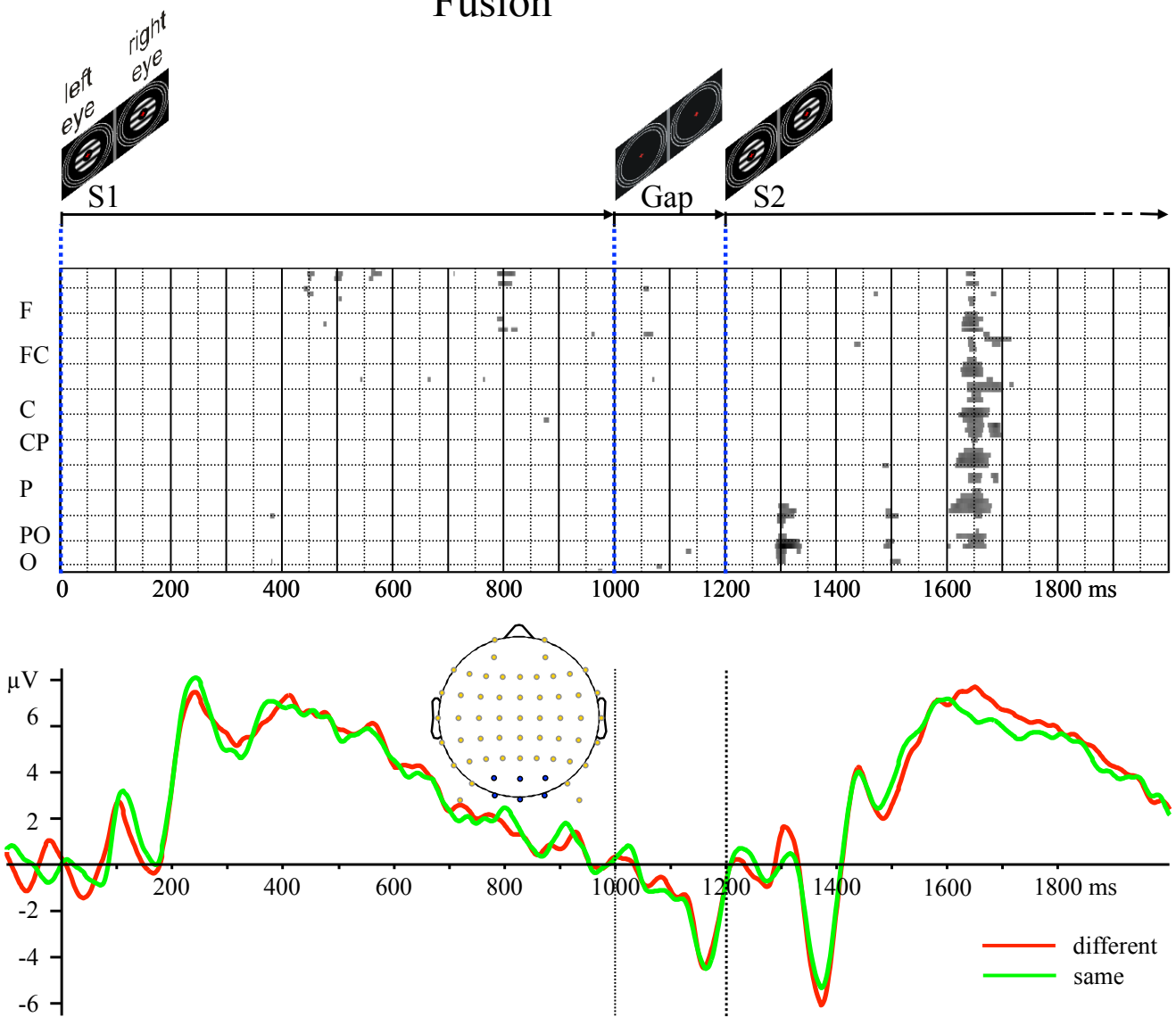

Supplement: Figure S2 — Version of Figure 1b showing an additional 800 ms of the second display of fused stimuli. Top panel: schematic representation of the stimuli as a function of time. Middle panel: t values for the difference in the voltage between trials when consciousness changed at the second display with those when it did not change. These are from all electrodes (arrayed on the y axis from frontal, F, at the top to occipital, O, at the bottom) as a function of time (on the x axis). There is no predictive activity in the first display. There are differences in the second display: higher voltages 100 ms and 450 ms after onset of different stimuli. Lower panel: Average voltages, ERPs, from clusters of six parieto-occipital and occipital (OP) electrodes, red for when consciousness changed and green for when it stayed the same. ERPs are essentially the same in the first display. In the second display, there are a bigger P1 and a bigger N1 when stimuli differed from the first display. There is also a late positivity from 300 ms to about 800 ms that is much bigger for different stimuli about 450 ms. We discuss these differences in Item S1. (PDF) [file pone.0076134.s002.pdf]
