# Supplementary material for: Predicting Visual Consciousness Electrophysiologically from Intermittent Binocular Rivalry
Source: PLoS One. 2013 Oct 4;8(10):e76134. doi: 10.1371/journal.pone.0076134 (PMC3790688; doi:10.1371/journal.pone.0076134)
Supplement: Table S1 — Two-factor ANOVAs of average voltage from parieo-occipital (PO, O) electrodes for Rivalry and Fusion conditions from 170 to 190 ms after onset of the rival stimuli. (DOCX) [file pone.0076134.s003.docx]

Table S1

*Two-factor ANOVAs of average voltage from parieo-occipital (PO, O) electrodes for Rivalry and Fusion conditions from 170 to 190 ms after onset of the rival stimuli*

| Source | *df* | *F* | *p* | *partial η^2^* |
| --- | --- | --- | --- | --- |
| RIVALRY |  |  |  |  |
| Percept (change vs no change) | 1,10 | 7.38 | **.022** | .42 |
| Site (left [PO3 & O1], middle [POz & Oz], right [PO4 & O2]) | 2,20 | 4.10 | .055 | .30 |
| Percept x Site | 2,20 | 0.48 | .270 | .12 |
|  |  |  |  |  |
| FUSION |  |  |  |  |
| Percept (change vs no change) | 1,10 | 0.42 | .530 | .04 |
| Site (left [PO3 & O1], middle [POz & Oz], right [PO4 & O2]) | 2,20 | 4.90 | **.043** | .33 |
| Percept x Site | 2,20 | 0.10 | .760 | .01 |
