# Supplementary material for: Predicting Visual Consciousness Electrophysiologically from Intermittent Binocular Rivalry
Source: PLoS One. 2013 Oct 4;8(10):e76134. doi: 10.1371/journal.pone.0076134 (PMC3790688; doi:10.1371/journal.pone.0076134)
Supplement: Table S2 — Two-factor ANOVAs of average voltage from central electrodes (fronto-central, FC, central, C, and centro-parietal, CP) for Rivalry and Fusion conditions from 380 to 420 ms after onset of the rival stimuli. (DOCX) [file pone.0076134.s004.docx]

Table S2

*Two-factor ANOVAs of average voltage from central electrodes (fronto-central, FC, central, C, and centro-parietal, CP) for Rivalry and Fusion conditions from 380 to 420 ms after onset of the rival stimuli*

| Source | *df* | *F* | *p* | *partial η^2^* |
| --- | --- | --- | --- | --- |
| RIVALRY |  |  |  |  |
| Percept (change vs no change) | 1,10 | 11.02 | **.008** | .52 |
| Site (left [FC1, C1, CP1], middle [FCz, Cz, CPz], PO right [FC2, C2, CP2) | 2,20 | 0.61 | .566 | .12 |
| Percept x Site | 2,20 | 2.35 | .151 | .34 |
|  |  |  |  |  |
| FUSION |  |  |  |  |
| Percept (change vs no change) | 1,10 | 0.47 | .508 | .05 |
| Site (left [FC1, C1, CP1], middle [FCz, Cz, CPz], PO right [FC2, C2, CP2) | 2,20 | 6.67 | **.017** | .60 |
| Percept x Site | 2,20 | 2.05 | .185 | .31 |
