# Supplementary material for: Predicting Visual Consciousness Electrophysiologically from Intermittent Binocular Rivalry
Source: PLoS One. 2013 Oct 4;8(10):e76134. doi: 10.1371/journal.pone.0076134 (PMC3790688; doi:10.1371/journal.pone.0076134)
Supplement: Item S1 — Discussion of activity in the second display. (DOCX) [file pone.0076134.s005.docx]

**Item S1: Discussion of Activity in the Second Display**

About 450 ms after the onset of the second display, deflections from rivalry trials in which consciousness changed after the gap are more negative than deflections from trials in which consciousness stayed the same after the gap (Figure S1). A similar negativity has recently been described for a Necker cube stimulus and interpreted as a residuum of a response-related Bereitschaftspotenzial [82].

The earliest differences in the second display of fusion conditions (Figure S2) occur about 100 ms after onset at the occipital and parieto-occipital electrodes. The ERP traces in the lower panel show that this difference is the well-known P1 component of the ERP [7]. The P1 is greater when visual consciousness changed in the second display. This may reflect neural adaptation [118]. During trials in which the stimuli were the same in the first and second displays, the same neurons would mediate visual consciousness. Adaptation would commence in the first display and would continue in the second display, reducing the P1 there. During trials in which the stimuli were different in the first and second displays, different neurons would mediate visual consciousness in the two displays. For example, although neurons mediating consciousness of vertical in the first display would adapt, neurons mediating consciousness of horizontal in the second display would not be adapted, yielding a vigorous P1.

There is also a major difference across many electrodes about 450 ms after onset of the second display in fusion conditions. This effect occurs at about the same time as the effect with rivalry stimuli, but with a different sign, positive rather than negative. A similar positivity has been reported from a disambiguated version of the Necker cube in a similar experimental paradigm [83]. It may well be that the fusion stimuli in the present study evoked a similar positivity obliterating the residuum of the Bereitschaftspotenzial, whereas the rivalry stimuli did not.

**New Reference**

118. Blakemore C, Campbell FW (1969) One the existence of neurones in the human visual system selectively sensitive to the orientation and size of retinal images. Journal of Physiology 203: 237-260.
